# Late posterior subluxation of rollable intraocular lens after an uneventful microphacoemulsification cataract surgery

**DOI:** 10.4103/0301-4738.64144

**Published:** 2010

**Authors:** Aditya S Kelkar, Jai A Kelkar, Shreekant B Kelkar, Aarofil I Shaikh

**Affiliations:** National Institute of Ophthalmology, Pune, India

**Figure 1:**
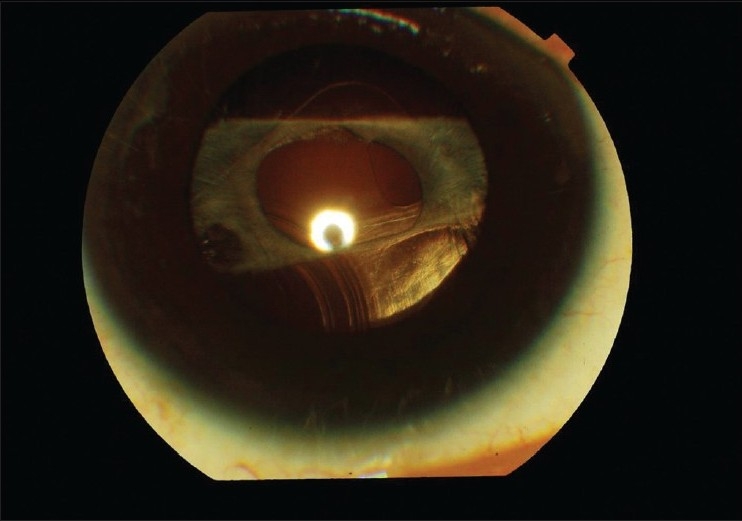
Anterior segment showing posterior subluxation of the rollable IOL and shrinkage of anterior capsule

**Figure 2:**
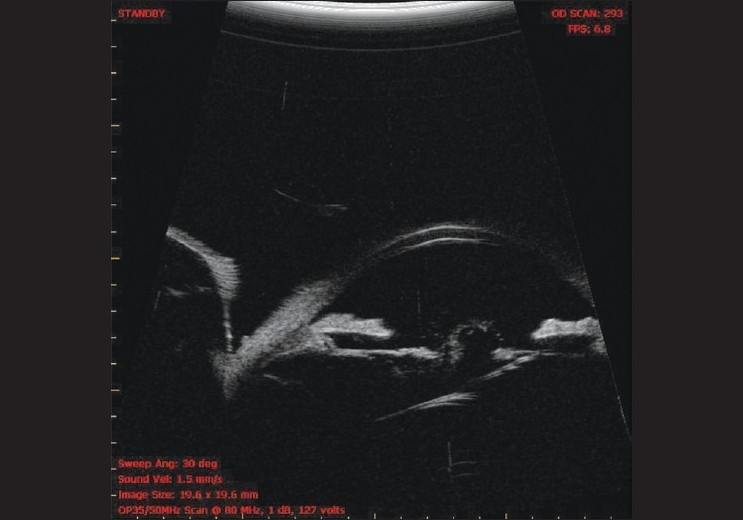
UBM demonstrating posterior subluxation of the rollable IOL with vitreous prolapsed in anterior chamber

Dear Editor,

We report a rare complication of late posterior subluxation of rollable intraocular lens (IOL) after an uneventful microphacoemulsification cataract surgery. A 60-year-old non-diabetic, non-hypertensive male patient was operated for microphacoemulsification, phaco chop technique for senile cataract in the right eye using 0.9 mm limbal incision which was enlarged to 1.5 mm and an acrylic rollable IOL (micriol, Eyeol, UK, optic size: 5.0 mm, overall length: 11.0 mm) was implanted in the bag. The patient regained 20/20 vision (on Snellen's chart) at the end of six weeks of follow-up and had no complaints. Then after three months the patient returned with decrease in vision in the right eye (20/200). The slit-lamp examination revealed posterior capsular opacification with shrinkage of anterior capsule. Patient underwent uneventful YAG capsulotomy (around 2 mm diameter). Patient regained 20/20 vision at two weeks follow-up. However, two months later, patient returned with painless progressive diminution of vision (finger counting at 2 meters) with glare and uniocular diplopia. There was no history of trauma. The slit-lamp examination [Fig. 1] and ultrasound biomicroscope [Fig. 2] demonstrated posterior subluxation of the rollable IOL with vitreous prolapsed in the anterior chamber. The patient was subjected to IOL exchange procedure and anterior vitrectomy. The rollable IOL was explanted through the scleral tunnel and a rigid polymethyl methacrylate (PMMA) lens was implanted in the sulcus. The patient regained 20/20 vision on the next follow-up.

Microincisional cataract surgery is a safe procedure with a very short learning period for an experienced cataract surgeon and rollable ultrathin IOLs eliminate the need for enlargement of corneal incision.[1] However, the rollable IOLs having plate haptics have their own set of problems. The posterior dislocation of the plate haptic IOL has been known to occur after Nd:YAG capsulotomy.[2] Even the anterior luxation of plate haptic IOL has been reported.[3,4]

It has been postulated that the shrinkage of the anterior capsule exerts centripetal forces on the ends of the plate haptics, causing the optic to move posteriorly and to exert pressure against the posterior capsule. If either the posterior or anterior capsule is disrupted by Nd:YAG laser treatment, the forces created by capsular contraction against the flexible lens may cause extension of radial tears and appear to be a substantial risk for further capsule-tearing, releasing the IOL into the vitreous cavity, even months later. The forces of capsular contraction can impart a spring-loading effect on plate haptic silicone lenses. Due to inadequate capsular adherence, these lenses are at risk of posterior dislocation from capsular rents following Nd:YAG laser treatment.[5]

It is important to have a close follow-up of patients with plate haptic rollable IOLs undergoing YAG capsulotomy. Patients should be warned of this rare possible complication. Vitrectomy and IOL exchange with rigid PMMA lens implantation in the sulcus seems to be effective in tackling this complication.
